# Clinical outcomes of COVID-19 and influenza in hospitalized children <5 years in the US

**DOI:** 10.3389/fped.2023.1261046

**Published:** 2023-09-11

**Authors:** Leah J. McGrath, Mary M. Moran, Tamuno Alfred, Maya Reimbaeva, Manuela Di Fusco, Farid Khan, Verna L. Welch, Deepa Malhotra, Alejandro Cane, Santiago M. C. Lopez

**Affiliations:** Pfizer Inc., New York, NY, United States

**Keywords:** COVID-19, disease severity, influenza, hospitalization outcomes, pediatric population

## Abstract

**Introduction:**

We compared hospitalization outcomes of young children hospitalized with COVID-19 to those hospitalized with influenza in the United States.

**Methods:**

Patients aged 0-<5 years hospitalized with an admission diagnosis of acute COVID-19 (April 2021-March 2022) or influenza (April 2019-March 2020) were selected from the PINC AI Healthcare Database Special Release. Hospitalization outcomes included length of stay (LOS), intensive care unit (ICU) admission, oxygen supplementation, and mechanical ventilation (MV). Inverse probability of treatment weighting was used to adjust for confounders in logistic regression analyses.

**Results:**

Among children hospitalized with COVID-19 (*n* = 4,839; median age: 0 years), 21.3% had an ICU admission, 19.6% received oxygen supplementation, 7.9% received MV support, and 0.5% died. Among children hospitalized with influenza (*n* = 4,349; median age: 1 year), 17.4% were admitted to the ICU, 26.7% received oxygen supplementation, 7.6% received MV support, and 0.3% died. Compared to children hospitalized with influenza, those with COVID-19 were more likely to have an ICU admission (adjusted odds ratio [aOR]: 1.34; 95% confidence interval [CI]: 1.21–1.48). However, children with COVID-19 were less likely to receive oxygen supplementation (aOR: 0.71; 95% CI: 0.64–0.78), have a prolonged LOS (aOR: 0.81; 95% CI: 0.75–0.88), or a prolonged ICU stay (aOR: 0.56; 95% CI: 0.46–0.68). The likelihood of receiving MV was similar (aOR: 0.94; 95% CI: 0.81, 1.1).

**Conclusions:**

Hospitalized children with either SARS-CoV-2 or influenza had severe complications including ICU admission and oxygen supplementation. Nearly 10% received MV support. Both SARS-CoV-2 and influenza have the potential to cause severe illness in young children.

## Introduction

COVID-19, caused by SARS-CoV-2, can lead to severe respiratory illness resulting in hospitalization and death (1, 2). Older age groups, particularly ≥65 years old, are more severely affected by COVID-19 as seen by higher rates of hospitalization, prolonged hospitalization, greater usage of mechanical ventilation (MV) support, and mortality (1–3). Several studies have shown that children and adolescents may also develop severe COVID-19 resulting in hospitalization with relatively high rates of admission to the intensive care unit (ICU) (2–11). Furthermore, but rarely, children and adolescents may suffer serious sequalae from COVID-19, such as multisystem inflammatory syndrome (MIS-C) and persistent complications (12–14). According to the COVID-19-Associated Hospitalization Surveillance Network (COVID-NET; contributors: >300 acute-care hospitals in 13 states in the US), as of June 2023, >7490 COVID-19-associated hospitalizations have been recorded among patients aged 0-<5 years (1).

Influenza, like COVID-19, can cause respiratory illness in children and adolescents; however, the differences in clinical disease severity caused by these pathogens mostly remains unknown, especially in younger age groups. In two US studies of relatively small pediatric populations, hospitalization outcomes were found to be similar among those diagnosed with COVID-19 and those diagnosed with influenza (15, 16). In a larger scale study conducted in the US that utilized data from COVID-NET and the Influenza Hospitalization Network (FluSurv-NET), Delahoy et al. (17) reported the COVID-19 hospitalization rate of 48.2 per 100 000 children <18 years of age (October 2020-September 2021) was higher than influenza-associated hospitalization rates for this age group during 2017–2018 (33.5/100,000), 2018–2019 (33.8/100,000), and 2019–2020 (41.7/100,000) influenza seasons.

Further study is warranted on the similarities and differences of COVID-19 and influenza illnesses in younger age groups, including infants, to provide better context for implementing public health measures and hospital-based care for pediatric populations, which may vary seasonally. Towards this goal, in this study using hospital administrative data from a nationally representative data source (18), we compared the clinical outcomes of young children (<5 years of age) hospitalized with COVID-19 to those hospitalized with influenza in the US.

## Methods

### Study design and data source

This study was a retrospective cohort analysis using the PINC AI™ (formerly known as Premier) Healthcare Database Special Release (PHD SR) (18). The PHD SR includes approximately 25% of annual US hospital admissions and is a hospital-based, service-level, all-payer database (18), and has been used previously for COVID-19 related studies (19–22). All data in the PHD SR are deidentified and compliant with the Health Insurance Portability and Accountability Act. This study was deemed exempt from Institutional Review Board review pursuant to the terms of the U.S. Department of Health and Human Service's Policy for Protection of Human Research Subjects as a category 4 exemption (Sterling IRB, Atlanta, GA).

### Study population

Two cohorts of hospitalized children aged <5 years were selected from the PHD SR (1): with COVID-19 between April 1, 2021, and March 31, 2022 and (2) with influenza virus infection between April 1, 2019 and March 31, 2020 (pre-pandemic period). Since influenza virus circulation was less predictable during the COVID-19 pandemic, the earlier index identification period for influenza was used to provide a more representative comparator cohort. Children were included if they had an ICD-10-CM diagnosis code for COVID-19 (U07.1) or influenza (J09.X, J10.X, or J11.X) in the primary or secondary position (i.e., all non-primary diagnosis positions) and flagged as “present on admission” (POA) (3). POA was required to ensure patients did not acquire the disease in the hospital and was also an attempt to limit incidental or asymptomatic infections (3). Subjects were excluded if they had: (1) both COVID-19 and influenza in the same admission, (2) influenza and were later re-admitted with COVID-19, (3) missing biological sex, and (4) infants who had an ICD-10-CM birth code (Z38.XX) during index inpatient admission, as it is possible that newborns with a COVID-19 code may have been assigned a COVID-19 code due to maternal SARS-CoV-2 infection at time of delivery. Only the first qualifying hospital admission during the respective study period was included in the analysis (i.e., no readmissions).

### Demographic characteristics and comorbid conditions

Demographic characteristics, including insurance type (as a proxy for socioeconomic status) and hospital location (to characterize regional distribution of the study population), and the prevalence of comorbid conditions (defined by ICD-10-CM codes) among study cohorts were evaluated during index hospitalization*.* Due to data source limitations, age is reported in yearly increments; monthly ages of infants <1 year were not available.

### Hospitalization outcomes

The hospitalization outcomes evaluated included length of hospitalization stay (LOS), admission to the intensive care unit (ICU), ICU LOS, usage and duration of oxygen supplementation, usage and duration of mechanical ventilatory (MV) support, and inpatient death. Duration outcomes such as LOS, ICU LOS, oxygen supplementation and MV duration were assigned as “prolonged” if the duration was longer than the median value for that outcome in the combined COVID-19 and influenza cohorts. Outcomes were identified overall (ages 0-<5) and stratified by age groups, 0–1 and 2-<5 years.

### Statistical analyses

Descriptive statistics were used to summarize patient demographic characteristics, prevalence of comorbid conditions, and unweighted hospitalization outcomes.

To account for confounding between the cohorts (e.g., differences in age, hospital location, etc.), we conducted logistic regression to generate propensity scores. In addition to the variables shown in Table 1, hospital census division and calendar month of admission were used in the regression analysis. Next, stabilized inverse probability of treatment weighting was applied to propensity scores to reweight patients in each cohort. An absolute standardized mean difference (SMD) <0.1 between cohorts was considered indicative of adequate balance. Weighted logistic regression outcome models were then used to evaluate the association of COVID-19 vs. influenza hospitalization with each hospitalization outcome. Adjusted odds ratios (aORs) with 95% confidence intervals (CIs) were reported. All statistical analyses were carried out in SAS 9.4 (SAS Institute; Cary, NC).

**Table 1:** Demographic characteristics and comorbid conditions of children aged 0-<5 years hospitalized with COVID-19 and influenza.

Characteristic	Before weighting		After weighting		SMD
	COVID-19 *n* = 4,839	Influenza *n* = 4,349	COVID-19 *n* = 4,910	Influenza *n* = 4,284	COVID-19 vs. Influenza
Age, year					
Mean (SD)	0.9 (1.2)	1.4 (1.4)	1.2 (1.4)	1.1 (1.3)	0.0211
Median (IQR)	0 (0–2)	1 (0–2)	1 (0–2)	1 (0–2)	
Age group, year, *n* (%)	* *	* *	* *	* *	0.0267
<1	2,672 (55.2)	1,592 (36.6)	2,224 (45.3)	1,985 (46.3)	
1	927 (19.2)	1,038 (23.9)	1,056 (21.5)	901 (21.0)	
2	533 (11.0)	685 (15.8)	646 (13.1)	569 (13.3)	
3	399 (8.2)	577 (13.3)	542 (11.0)	467 (10.9)	
4	308 (6.4)	457 (10.5)	442 (9.0)	362 (8.5)	
Sex, *n* (%)	* *	* *	* *	* *	0.0054
Male	2,701 (55.8)	2,499 (57.5)	2,774 (56.5)	2,431 (56.8)	
Female	2,138 (44.2)	1,850 (42.5)	2,136 (43.5)	1,852 (43.2)	
Race/ethnicity^a^, *n* (%)					0.0248
White non-hispanic	1,662 (34.3)	1,182 (27.2)	1,495 (30.5)	1,286 (30.0)	
Black non-hispanic	795 (16.4)	754 (17.3)	835 (17.0)	728 (17.0)	
Other non-hispanic	303 (6.3)	265 (6.1)	305 (6.2)	252 (5.9)	
White hispanic	808 (16.7)	689 (15.8)	817 (16.6)	721 (16.8)	
Black hispanic	46 (1.0)	30 (0.7)	40 (0.8)	38 (0.9)	
Other hispanic	465 (9.6)	426 (9.8)	485 (9.9)	423 (9.9)	
Asian hispanic/non-hispanic	117 (2.4)	116 (2.7)	121 (2.5)	116 (2.7)	
Unknown	643 (13.3)	887 (20.4)	810 (16.5)	720 (16.8)	
Insurance type, *n* (%)					0.0476
Medicaid	3,318 (68.6)	2,997 (68.9)	3,414 (69.5)	3,017 (70.4)	
Commercial	1,147 (23.7)	1,066 (24.5)	1,147 (23.4)	1,011 (23.6)	
Medicare	5 (0.1)	5 (0.1)	5 (0.1)	5 (0.1)	
Other	289 (6.0)	146 (3.4)	219 (4.5)	155 (3.6)	
Uninsured	80 (1.7)	135 (3.1)	125 (2.5)	97 (2.3)	
Hospital location, *n* (%)					0.0185
Urban	4,591 (94.9)	3,965 (91.2)	4,557 (92.8)	3,996 (93.3)	
Rural	248 (5.1)	384 (8.8)	353 (7.2)	288 (6.7)	
Comorbid conditions, *n* (%)					
Immunocompromised^b^	772 (16.0)	551 (12.7)	709 (14.4)	605 (14.1)	0.0088
Diabetes	28 (0.6)	23 (0.5)	27 (0.5)	24 (0.6)	−0.0021
Obesity/overweight	25 (0.5)	21 (0.5)	27 (0.5)	26 (0.6)	−0.0092
Hypertension	76 (1.6)	56 (1.3)	72 (1.5)	70 (1.6)	−0.0127
Neurological disease	334 (6.9)	222 (5.1)	305 (6.2)	261 (6.1)	0.0048
Asthma/reactive airway disease	360 (7.4)	653 (15.0)	596 (12.1)	503 (11.7)	0.0123
Down Syndrome/chromosomal anomaly	75 (1.5)	57 (1.3)	66 (1.3)	58 (1.4)	−0.0017
Metabolic disease	32 (0.7)	30 (0.7)	32 (0.6)	26 (0.6)	0.0055
Sickle cell disease	89 (1.8)	96 (2.2)	103 (2.1)	86 (2.0)	0.0064
Congenital heart condition	25 (0.5)	14 (0.3)	21 (0.4)	17 (0.4)	0.0052
Congenital lung condition	10 (0.2)	20 (0.5)	16 (0.3)	14 (0.3)	0.0017
Autoimmune disease	30 (0.6)	18 (0.4)	26 (0.5)	21 (0.5)	0.0031
Transplant (bone marrow/organ)	29 (0.6)	12 (0.3)	22 (0.5)	17 (0.4)	0.0069
Disability^c^	47 (1.0)	33 (0.8)	40 (0.8)	34 (0.8)	0.0033

IQR, interquartile range; SD, standard deviation; SMD, standardized mean difference.

^a^ Unknown refers to either one of, or both, race and ethnicity are unknown.

^b^ Immunocompromised conditions included HIV/AIDS, solid malignancy, bone marrow transplant, organ transplant, rheumatologic/other inflammatory condition, primary immunodeficiency, chronic kidney disease/end stage renal disease, and other immune conditions.

^c^ Includes neurologic, neurodevelopmental, intellectual, physical, vision or hearing impairment.

## Results

### Study population

**Figure 1:**
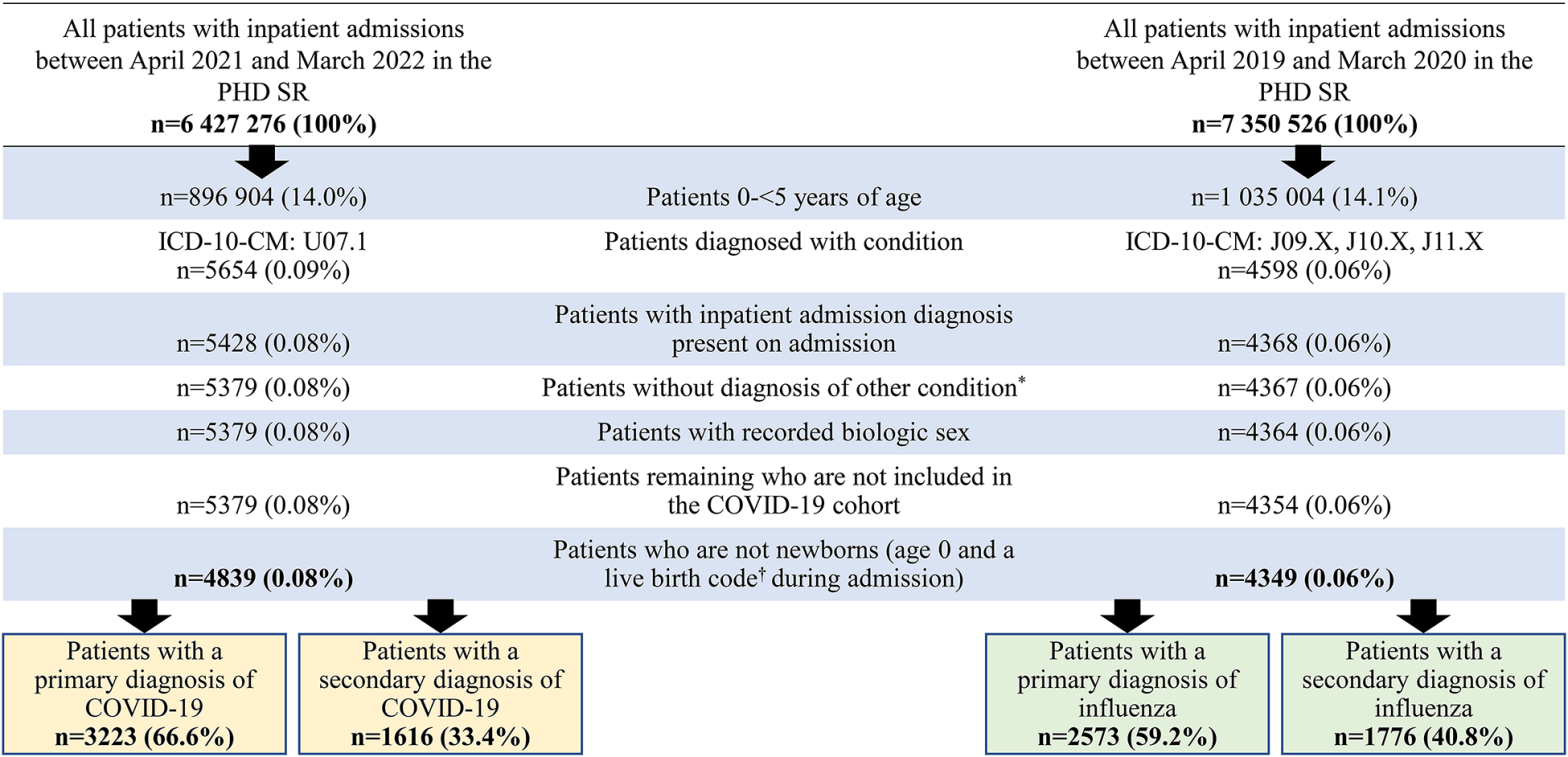
Patient selection process. *For example, for the COVID-19 cohort, excluded hospitalizations with an influenza diagnosis. **^†^**ICD-10-CM: Z38.XX.

The patient selection process for each cohort is shown in Figure 1. Of the total inpatient admissions recorded in the PHD SR between April 2021 and March 2022, 896,904 (14.0%) were children aged <5 years and 5,428 (0.08%) had a COVID-19 diagnosis POA. There were 4,839 patients remaining after study exclusion criteria were applied.

Of the total inpatient admissions recorded in the PHD SR between April 2019 and March 2020, 1,035,004 (14.1%) were children aged <5 years and 4,368 (0.06%) had a confirmed influenza diagnosis POA. There were 4,349 (0.06%) patients remaining after study exclusion criteria were applied.

### Characteristics of unweighted study cohorts

Demographic characteristics and prevalence of comorbid conditions are shown in Table 1. The median (IQR) age of the cohort hospitalized with COVID-19, was 0 (0–2) years; COVID-19 disproportionally impacted infants ≤1 year compared with influenza (74.4% vs. 60.5%, respectively).

Among the COVID-19 cohort, the majority of children were male (55.8%). Approximately one-third (34.3%) were White non-Hispanic, 27.3% were Hispanic, and 16.4% were Black non-Hispanic. Over two-thirds (68.6%) were Medicaid insured. Among those hospitalized with COVID-19, 16.0% had immunocompromising conditions. Other more prevalent comorbid conditions included asthma/reactive airway disease (7.4%) and neurological disease (6.9%).

Children hospitalized with influenza were slightly older with a median age of 1 (0–2) years compared with the COVID-19 cohort. Similar to the hospitalized COVID-19 cohort, the majority of children were male (57.5%). Just over one-quarter (27.2%) were White non-Hispanic, 26.3% were Hispanic, and 17.3% were Black non-Hispanic. Over two-thirds (68.9%) were Medicaid insured. Fewer children with influenza had an immunocompromising condition (12.7%). The prevalence of asthma/reactive airway disease (15.0%) was approximately twice that observed in the COVID-19 cohort.

Generally similar demographic distributions were observed for both cohorts in the 0–1- and 2-<5-year age groups (Supplementary Tables S1,S2).

### Descriptive index hospitalization outcomes

The distribution of unadjusted hospitalization outcomes for each disease cohort, overall and stratified by age groups are shown in Table 2.

**Table 2:** Descriptive hospitalization outcomes for children with COVID-19 and influenza, overall and stratified by age groups.

Outcome	0-<5 Years of Age		0–1 Year of Age		2-<5 Years of Age	
	COVID-19 *n* = 4,839	Influenza *n* = 4,349	COVID-19 *n* = 3,599	Influenza *n* = 2,630	COVID-19 *n* = 1,240	Influenza *n* = 1,719
**Hospitalization LOS, days**						
Mean (SD)	3.7 (7.2)	3.4 (5.3)	3.7 (7.5)	3.5 (5.7)	3.8 (6.3)	3.3 (4.5)
Median (IQR)	2 (1–4)	2 (1–4)	2 (1–3)	2 (1–4)	2 (1–4)	2 (1–3)
**ICU admission,** ***n*** **(%)**	1,032 (21.3)	758 (17.4)	750 (20.8)	479 (18.2)	282 (22.7)	279 (16.2)
ICU LOS, days						
Mean (SD)	4.9 (9.5)	4.6 (6.9)	5.1 (10.2)	4.9 (7.6)	4.5 (7.3)	4.1 (5.6)
Median (IQR)	2 (1–4)	3 (1–5)	2 (1–4)	3 (1–5)	2 (1–4)	2 (1–5)
**Oxygen supplementation, *n* (%)**	947 (19.6)	1,162 (26.7)	713 (19.8)	767 (29.2)	234 (18.9)	395 (23.0)
Duration of oxygen supplementation, days						
Mean (SD)	3.5 (5.1)	3.2 (4.1)	3.6 (5.3)	3.3 (4.5)	3.4 (4.0)	3.0 (3.2)
Median (IQR)	2 (1–4)	2 (1–4)	2 (1–4)	2 (1–4)	2 (1–4)	2 (1–4)
**MV support, *n* (%)**	383 (7.9)	332 (7.6)	272 (7.6)	215 (8.2)	111 (9.0)	117 (6.8)
Duration of MV support, days						
Mean (SD)	6.7 (11.5)	4.9 (6.8)	6.8 (11.8)	5.1 (6.5)	6.4 (10.7)	4.5 (7.4)
Median (IQR)	3 (1–8)	3 (1–6)	3 (1–8)	3 (1–6)	2 (1–7.5)	2.5 (1–5)
**Inpatient death, *n* (%)**	25 (0.5)	14 (0.3)	18 (0.5)	9 (0.3)	7 (0.6)	5 (0.3)

ICU, intensive care unit; IQR, interquartile range; LOS, length of stay; MV, mechanical ventilation; SD, standard deviation.

#### COVID-19

Among the overall COVID-19 cohort, the median hospitalization LOS was 2 (1–4) days. A total of 1,032 patients (21.3%) were admitted to the ICU for a median LOS of 2 (1–4) days. Oxygen supplementation was administered to 947 (19.6%) patients for a median duration of 2 (1–4) days and MV support was received by 383 (7.9%) for a median of 3 (1–8) days. Inpatient death occurred among 0.5% of COVID-19 cohort, and all deaths occurred among children admitted to the ICU and on MV support. Compared to the overall study population, outcomes were generally similar for the age groups of 0–1 and 2-<5 years. However, disease severity was greater among patients aged 2-<5 years compared to those aged 0–1 year; more were admitted to the ICU (22.7% vs. 20.8% in the 0–1 cohort), a larger proportion required MV support (9.0% vs. 7.6% in the 0–1 cohort), and a slightly greater proportion suffered inpatient death (*n* = 7; 0.6% vs. *n* = 18; 0.5% in the 0–1 cohort).

#### Influenza

Among the overall influenza cohort, the median LOS for hospitalization was 2 (1–4) days and 758 (17.4%) children were admitted to the ICU. A total of 1,162 (26.7%) patients received oxygen supplementation, which was higher than that observed in the hospitalized COVID-19 cohort, but the average duration was shorter. A total of 332 (7.6%) children received MV support and the rate of inpatient death was lower than the COVID-19 cohort (0.3%). In contrast to the hospitalized COVID-19 cohort, patients in the influenza cohort aged 0–1 year had greater disease severity than those aged 2-<5 years; more were admitted to the ICU (18.2% vs. 16.2% in the 2-<5 cohort) for a longer LOS (median: 3 vs. 2 days in the 2-<5 cohort), and larger proportions required oxygen supplementation (29.2% vs. 23.0% in the 2-<5 cohort) and MV support (8.2% vs. 6.8% in the 2-<5 cohort). Inpatient death rates were similar for patients aged 0–1 year and those aged 2-<5 years.

### Weighted association of COVID-19 versus influenza hospitalization with hospitalization outcomes

**Figure 2:**
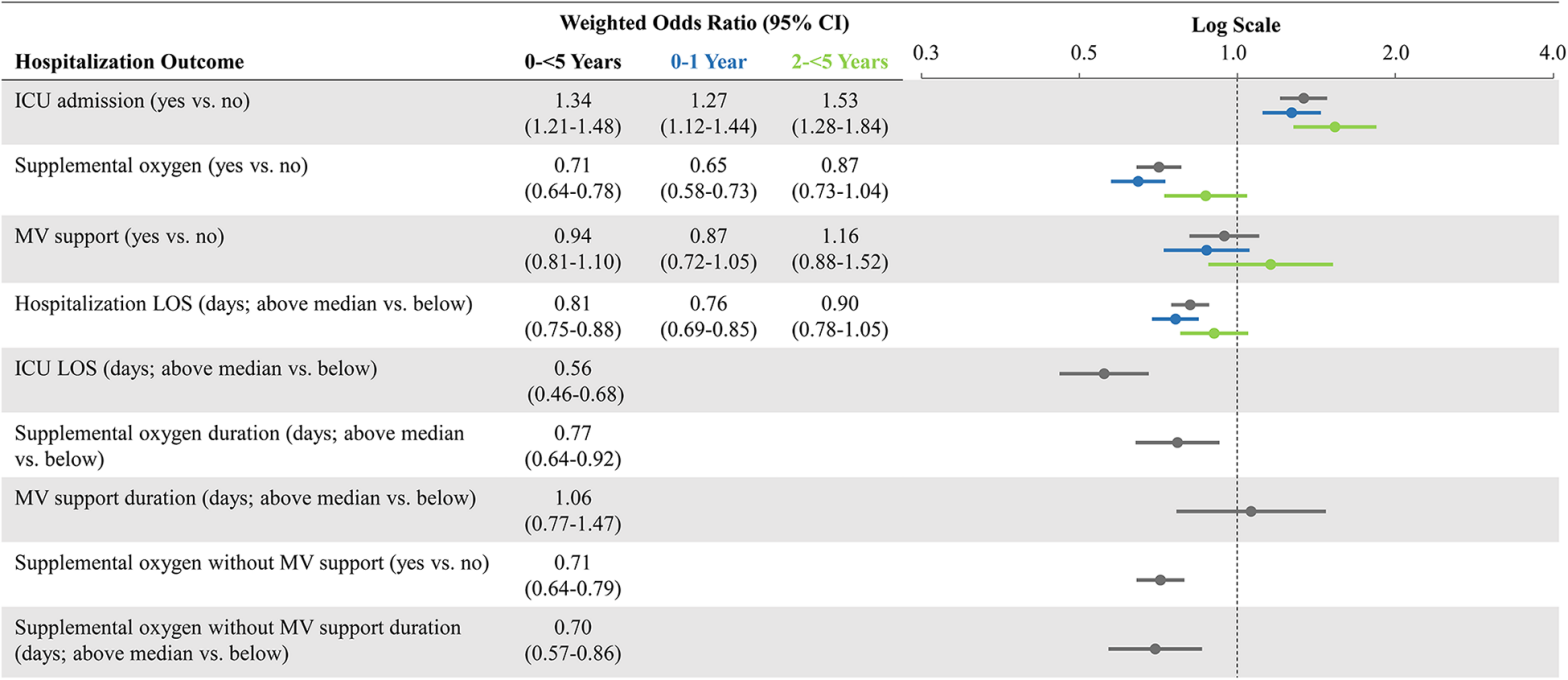
Weighted odds ratios of hospitalization outcomes* comparing COVID-19 versus influenza, overall and stratified by age groups. CI, confidence interval; ICU, intensive care unit; LOS, length of stay; MV, mechanical ventilation; vs.: versus. *Prolonged outcomes other than hospitalization LOS, are not shown in age subgroups due to small number of patients with those events and greater evidence of after-weighting covariate imbalance.

After weighting, demographic and clinical characteristics were well balanced between the pediatric cohorts (SMDs reported in Table 1). Weighted ORs of hospitalization outcomes for the overall study cohorts and stratified by age groups are shown in Figure 2. Compared to patients 0-<5 years of age hospitalized with influenza, those hospitalized with COVID-19 had a greater likelihood of ICU admission (aOR: 1.34; 95% CI: 1.21–1.48) but had a lower likelihood of receiving oxygen supplementation (aOR: 0.71; 95% CI: 0.64–0.78). The likelihood of receiving MV support was similar for both cohorts. Compared to patients hospitalized with influenza, those hospitalized with COVID-19 had lower likelihoods for prolonged LOS for their hospitalization (aOR: 0.81; 95% CI: 0.75–0.88) and in the ICU (aOR: 0.56; 95% CI: 0.46–0.68); they were also less likely to have prolonged usage of supplemental oxygen (aOR: 0.77; 95% CI: 0.64–0.92). Patients aged 0–1 year had generally similar findings as the overall cohort (0-<5 years of age). However, patients aged 2-<5 years hospitalized with COVID-19 had even greater odds for ICU admission (aOR: 1.53; 95% CI: 1.28–1.84) compared to the hospitalized influenza cohort aged 2-<5 years. However, likelihoods of supplemental oxygen usage and MV support, as well as likelihood for prolonged index hospitalization LOS, were more similar (Figure 2).

## Discussion

In this study of nearly 10,000 hospitalized children <5 years of age in the US, both COVID-19 and influenza caused clinically severe disease, with 21.3% and 17.4% of children, respectively, admitted to the ICU, 19.6% and 26.7%, respectively, received oxygen supplementation, and 7.9% and 7.6%, respectively, received MV support. COVID-19 deaths occurred among children admitted to the ICU and on MV support. COVID-19 affected a greater proportion of very young children (0–1 year of age) compared to influenza (74.4% compared with 60.5% with influenza). Although we used different age categories in our study, our findings are consistent with data recorded in COVID-NET (June 2023), which shows a higher number of cumulative COVID-19-associated hospitalizations among infants 0–6 months of age (1708.8/100,000) compared to the overall age group of 0-<5 years (395.8/100,000) (1).

Our study yielded similar results to other previously published studies. Delahoy et al. (17) was based on COVID-NET and FluSurv data and reported similar hospitalization outcomes for children aged 0-<5years; median hospitalization LOS was 2 days for both COVID-19- and influenza-associated hospitalizations, ICU admission proportions were 23.5% and 20.7%, respectively, MV support proportions were 5.9% and 5.4%, respectively, and inpatient death occurred in 0.8% and 0.5%, respectively. Compared to our study findings, the proportion of children with ICU admission was slightly higher and MV support was slightly lower, but the proportions were directionally consistent. These differences are possibly due to variations in calendar period covered in each study. Marks et al. (11) also published generally similar results among hospitalized children aged 0-<5 years with laboratory confirmed COVID-19 (COVID-NET data); although, in our study, the frequency of ICU admission was to some extent lower than reported in Marks et al. (11) (21.3% vs. 23.9%, respectively), median hospitalization LOS was longer (2 vs. 1.5 days, respectively), and frequency of MV support was higher (7.9% vs. 5.7%, respectively). Across all 3 studies, inpatient deaths among patients aged 0-<5 years were relatively rare (<1%) for COVID-19-associated hospitalizations (11, 17). A recent publication by Halasa et al. (16), in which life-threatening complications of influenza and COVID-19 were compared in children <21 years old, showed that critically ill children with COVID-19 had longer LOS in the hospital and ICU compared with influenza, particularly for children 2 through 4 years of age (16). Our results suggest the opposite relationship with higher odds of prolonged hospital LOS and ICU stay for children with influenza compared with COVID-19 in the overall cohort (0 through 4 years of age, Figure 2). These differences are likely driven by the time periods used to identify COVID-19 hospitalizations; Halasa et al. (16) included children hospitalized in the early period of the pandemic (March through December 2020), while our study focused on a later period covering Delta and Omicron waves. Additional differences in study design could also have impacted the results, including differences in the underlying source population; Halasa et al. (16) included only critically ill individuals and older aged children. The findings of our study on hospitalization outcomes of young children hospitalized with influenza showed higher frequency of ICU admission than observed for both historical seasonal influenza (2004–2009) and the H1N1 pandemic strain, but similar length of ICU stay (23–25). Our reported hospitalization LOS among the influenza cohort is similar to that previously reported among children hospitalized with influenza (26, 27).

After balancing differences in characteristics of the pediatric cohorts in this study, patients aged 0-<5 years hospitalized with COVID-19 were more likely to be admitted to the ICU than those hospitalized with influenza; however, they were less likely to have a prolonged hospitalization and ICU stay. Other hospitalization outcomes indicative of disease severity were generally similar or less likely for patients hospitalized with COVID-19 vs. influenza. The association of COVID-19 hospitalization with ICU admission may be attributed to several reasons including increased disease severity for children with COVID-19, closer monitoring due to uncertainty in disease outcomes in the pediatric population, fewer approved therapeutics for this age group compared with older children and adults, and scant and changing recommendations to guide the clinical management of pediatric populations, especially early in the pandemic (28). In addition, SARS-CoV-2 recommended isolation protocol suggests airborne precautions requiring negative room pressure, which might be only available in an ICU setting. The magnitude of the observed hospitalization outcomes in this study and the comparison of these outcomes across COVID-19- and influenza-associated hospitalizations underscores that both viral pathogens can cause severe clinical disease in young children.

We observed that disease severity was greater among COVID-19 patients aged 2-<5 years compared to those aged 0–1 year; more were admitted to the ICU (22.7% vs. 20.8% in the 0–1 cohort) and a larger proportion required MV support (9.0% vs. 7.6% in the 0–1 cohort). These differences could be in part due to maternal COVID-19 immunization status, since studies have shown that maternal COVID-19 immunization is associated with a reduced risk of hospitalization among young infants (29–31). Furthermore, infants 0–1 year of age might have been hospitalized due to an abundance of caution and close clinical monitoring for this new disease.

For decades, influenza has been known to cause severe disease that can result in hospitalization, especially in younger children (32). The US Advisory Committee on Immunization Practices recommends universal influenza vaccination, including children 6 months of age and older, particularly for those younger than 2 years of age and those with underlying medical conditions, such as asthma, neurologic disease, and diabetes (33, 34). During the 2019–2020 influenza season in the US, among children aged 6 months to <5 years (estimated vaccination coverage: 75.5%), the Centers for Disease Control and Prevention estimated that >930,000 medical visits, >9600 hospitalizations, and approximately 82 deaths were avoided with influenza vaccination (35). Our results suggest that COVID-19 has a similar level of disease severity. While there are COVID-19 vaccines recommended for children starting at 6 months of age that were authorized in June 2022 by the Food and Drug Administration (36), as of June 2023 only 2.00 million children <5 years of age (∼11% of the 19 million children in this age group) had received ≥1 COVID-19 mRNA vaccine dose(s) (37–39). Further public health measures may be needed to increase the uptake of COVID-19 mRNA vaccines among young children so that they receive adequate protection with the hope of averting hospitalization, especially during the winter months in which both influenza and COVID-19 activity may peak.

The findings of this study should be interpreted in the context of its limitations. First, some cases may have been hospitalized for reasons other than COVID-19 or influenza. To limit this occurrence, we included a diagnosis of COVID-19 or influenza that was POA. Second, other pediatric outcomes, such as MIS-C and long-term COVID-19 and influenza complications, were not examined as data after discharge were not available. Further study with other data sources that better capture outpatient medical visits is warranted. Third, we did not assess COVID-19 and influenza symptomatology and thus, this study lacked granularity on clinical presentation. Fourth, age was reported in yearly increments; therefore, we were unable to look at monthly ages of infants <1 year, which is needed to characterize populations eligible for vaccination. However, our COVID-19 cohort study period (April 2021-March 2022) occurred before the COVID-19 vaccine authorization for children ≥6 months—4 years (36). Fifth, information from other facilities for children who were transferred was not available. Sixth, ICU admission was considered as a surrogate of severity of disease; however, children hospitalized with COVID-19 may have been admitted to the ICU for close monitoring or need for isolation but not necessarily due to severe disease. Seventh, the study encompasses the initial wave of Omicron but not the subsequent sub-lineages. All subjects that required MV or died were in the ICU setting. Eight, information on maternal COVID-19 and influenza vaccination or general immunization history for the children in this study population were not captured in the data source. Therefore, we did not evaluate the impact of maternal and child influenza immunization on disease severity. Additionally, we had no data on antiviral therapy (e.g., oseltamivir, remdesivir), which is generally administered in the outpatient setting. Finally, the PHD SR is representative of approximately one quarter of the hospitalizations that occur annually in the US and all types of insurers were included, but our study findings may not generalize to the entire US or regions not represented in the data source.

## Conclusions

In this large retrospective cohort analysis of hospitalized children <5 years of age in the US, both COVID-19 and influenza caused clinically severe disease, with substantial portions of those hospitalized being admitted to the ICU, receiving oxygen supplementation, and receiving MV support. Relative to influenza, COVID-19 disproportionally affected infants and toddlers 0–1 year of age. The findings of this study emphasize the importance of prevention measures such as vaccination against both SARS-CoV-2 and influenza in young children.

## Data Availability

The original contributions presented in the study are included in the article/**Supplementary Material**, further inquiries can be directed to the corresponding author.
