# Supplementary material for: Clinical outcomes of COVID-19 and influenza in hospitalized children <5 years in the US
Source: Front Pediatr. 2023 Sep 11;11:1261046. doi: 10.3389/fped.2023.1261046 (PMC10518399; doi:10.3389/fped.2023.1261046)
Supplement: Supplementary file 1 [file Table1.pdf]

Supplementary Material

Clinical Outcomes of COVID-19 and Influenza in Hospitalized Children <5 Years in the US

Leah J McGrath,[1] PhD; Mary M Moran,[1] MD; Tamuno Alfred,[1]PhD; Maya Reimbaeva,[1] MS; Manuela Di Fusco,[1] PhD; Farid Khan,[1] PhD; Verna L Welch,[1] PhD, MPH; Deepa Malhotra,[1] MBA, MS; Alejandro Cane,[1] MD, PhD; Santiago MC Lopez,[1*] MD

Correspondence: Santiago.Lopez@pfizer.com

Supplementary Table 1. Demographic characteristics and comorbid conditions of children aged 0-1 year hospitalized with COVID-19 and influenza

Characteristic	Before Weighting COVID-19 n=3599	Before Weighting Influenza n=2630	After Weighting COVID-19 n=3650	After Weighting Influenza n=2595	SMD COVID-19 vs. Influenza
**Age, y**					
Mean (SD)	0.3 (0.4)	0.4 (0.5)	0.3 (0.5)	0.3 (0.5)	0.0334
Median (IQR)	0 (0-1)	0 (0-1)	0 (0-1)	0 (0-1)	
**Age group, y, n (%)**					-0.0334
<1	2672 (74.2)	1592 (60.5)	2456 (67.3)	1786 (68.8)	
1	927 (25.8)	1038 (39.5)	1194 (32.7)	808 (31.2)	
**Sex, n (%)**					0.0103
Male	2024 (56.2)	1526 (58.0)	2091 (57.3)	1499 (57.8)	
Female	1575 (43.8)	1104 (42.0)	1560 (42.7)	1095 (42.2)	
**Race/ethnicity,[*] n (%)**					0.0322
White Non-Hispanic	1246 (34.6)	658 (25.0)	1103 (30.2)	779 (30.0)	
Black Non-Hispanic	575 (16.0)	452 (17.2)	603 (16.5)	418 (16.1)	
Other Non-Hispanic	219 (6.1)	172 (6.5)	231 (6.3)	154 (5.9)	
White Hispanic	601 (16.7)	445 (16.9)	618 (16.9)	458 (17.6)	
Black Hispanic	25 (0.7)	15 (0.6)	22 (0.6)	14 (0.5)	
Other Hispanic	351 (9.8)	270 (10.3)	361 (9.9)	264 (10.2)	
Asian Hispanic/Non-Hispanic	82 (2.3)	57 (2.2)	82 (2.3)	64 (2.5)	
Unknown	500 (13.9)	561 (21.3)	631 (17.3)	445 (17.1)	
**Insurance type, n (%)**					0.0495
Medicaid	2498 (69.4)	1951 (74.2)	2620 (71.8)	1895 (73.0)	
Commercial	827 (23.0)	527 (20.0)	784 (21.5)	551 (21.3)	
Medicare	4 (0.1)	2 (0.1)	4 (0.1)	3 (0.1)	
Other	212 (5.9)	70 (2.7)	159 (4.4)	90 (3.5)	
Uninsured	58 (1.6)	80 (3.0)	83 (2.3)	55 (2.1)	
**Hospital location, n (%)**					0.0246
Urban	3412 (94.8)	2396 (91.1)	3384 (92.7)	2422 (93.3)	
Rural	187 (5.2)	234 (8.9)	266 (7.3)	173 (6.7)	
**Comorbid conditions, n (%)**					
Immunocompromised[†]	449 (12.5)	283 (10.8)	438 (12.0)	296 (11.4)	0.0185
Diabetes	2 (0.1)	6 (0.2)	2 (0.1)	4 (0.1)	-0.0266
Obesity/overweight	9 (0.3)	8 (0.3)	12 (0.3)	6 (0.2)	0.0148
Hypertension	46 (1.3)	30 (1.1)	44 (1.2)	32 (1.2)	-0.0022
Neurological disease	131 (3.6)	76 (2.9)	117 (3.2)	86 (3.3)	-0.0056
Asthma/reactive airway disease	134 (3.7)	236 (9.0)	243 (6.7)	160 (6.2)	0.0192
Down syndrome/ chromosomal anomaly	54 (1.5)	28 (1.1)	47 (1.3)	35 (1.4)	-0.0062
Metabolic disease	22 (0.6)	9 (0.3)	18 (0.5)	11 (0.4)	0.0097
Sickle cell disease	49 (1.4)	50 (1.9)	62 (1.7)	43 (1.7)	0.0041
Congenital heart condition	18 (0.5)	8 (0.3)	14 (0.4)	8 (0.3)	0.0170

Congenital lung condition	9 (0.3)	13 (0.5)	14 (0.4)	9 (0.4)	0.0058
Autoimmune disease	4 (0.1)	2 (0.1)	3 (0.1)	2 (0.1)	0.0038
Transplant (bone marrow/organ)	7 (0.2)	2 (0.1)	5 (0.1)	2 (0.1)	0.0151
Disability[‡]	19 (0.5)	10 (0.4)	16 (0.4)	11 (0.4)	0.0007

IQR, interquartile range; SD, standard deviation; SMD, standardized mean difference.

[*] Unknown refers to either one of, or both, race and ethnicity are unknown.

[†] Immunocompromised conditions included HIV/AIDS, solid malignancy, bone marrow transplant, organ transplant, rheumatologic/other inflammatory condition, primary immunodeficiency, chronic kidney disease/end stage renal disease, and other immune conditions.

[‡] Includes neurologic, neurodevelopmental, intellectual, physical, vision or hearing impairment.

Supplementary Table 2. Demographic characteristics and comorbid conditions of children aged 2-<5 years hospitalized with COVID-19 and influenza

Characteristic	Before Weighting COVID-19 n=1240	Before Weighting Influenza n=1719	After Weighting COVID-19 n=1237	After Weighting Influenza n=1701	SMD COVID-19 vs. Influenza
**Age, y**					
Mean (SD)	2.8 (0.8)	2.9 (0.8)	2.9 (0.8)	2.8 (0.8)	0.0199
Median (IQR)	3 (2-3)	3 (2-4)	3 (2-4)	3 (2-4)	
**Age group, y, n (%)**					0.0208
2	533 (43.0)	685 (39.8)	495 (40.0)	697 (41.0)	
3	399 (32.2)	577 (33.6)	415 (33.5)	564 (33.1)	
4	308 (24.8)	457 (26.6)	328 (26.5)	440 (25.9)	
**Sex, n (%)**					-0.0074
Male	677 (54.6)	973 (56.6)	682 (55.1)	932 (54.7)	
Female	563 (45.4)	746 (43.4)	555 (44.9)	770 (45.3)	
**Race/ethnicity,[*] n (%)**					0.0561
White Non-Hispanic	416 (33.5)	524 (30.5)	393 (31.8)	525 (30.9)	
Black Non-Hispanic	220 (17.7)	302 (17.6)	216 (17.5)	309 (18.2)	
Other Non-Hispanic	84 (6.8)	93 (5.4)	74 (6.0)	96 (5.7)	
White Hispanic	207 (16.7)	244 (14.2)	195 (15.7)	253 (14.9)	
Black Hispanic	21 (1.7)	15 (0.9)	15 (1.2)	25 (1.5)	
Other Hispanic	114 (9.2)	156 (9.1)	118 (9.6)	157 (9.2)	
Asian Hispanic/Non-Hispanic	35 (2.8)	59 (3.4)	38 (3.0)	58 (3.4)	
Unknown	143 (11.5)	326 (19.0)	187 (15.1)	277 (16.3)	
**Insurance type, n (%)**					0.0427
Medicaid	820 (66.1)	1046 (60.8)	794 (64.2)	1090 (64.1)	
Commercial	320 (25.8)	539 (31.4)	349 (28.2)	497 (29.2)	
Medicare	1 (0.1)	3 (0.2)	1 (0.1)	2 (0.1)	
Other	77 (6.2)	76 (4.4)	59 (4.8)	70 (4.1)	
Uninsured	22 (1.8)	55 (3.2)	34 (2.7)	42 (2.5)	
**Hospital location, n (%)**					-0.0085
Urban	1179 (95.1)	1569 (91.3)	1152 (93.2)	1582 (93.0)	
Rural	61 (4.9)	150 (8.7)	84 (6.8)	120 (7.0)	
**Comorbid conditions, n (%)**					
Immunocompromised[†]	323 (26.0)	268 (15.6)	239 (19.3)	317 (18.6)	0.0184
Diabetes	26 (2.1)	17 (1.0)	18 (1.5)	24 (1.4)	0.0041
Obesity/overweight	16 (1.3)	13 (0.8)	13 (1.0)	24 (1.4)	-0.0325
Hypertension	30 (2.4)	26 (1.5)	24 (1.9)	38 (2.3)	-0.0212
Neurological disease	203 (16.4)	146 (8.5)	149 (12.0)	198 (11.6)	0.0124
Asthma/reactive airway disease	226 (18.2)	417 (24.3)	279 (22.6)	392 (23.0)	-0.0107
Down syndrome/ chromosomal anomaly	21 (1.7)	29 (1.7)	21 (1.7)	26 (1.5)	0.0128
Metabolic disease	10 (0.8)	21 (1.2)	12 (1.0)	17 (1.0)	-0.0001

Sickle cell disease	40 (3.2)	46 (2.7)	36 (2.9)	46 (2.7)	0.0162
Congenital heart condition	7 (0.6)	6 (0.3)	6 (0.5)	9 (0.5)	-0.0054
Congenital lung condition	1 (0.1)	7 (0.4)	2 (0.2)	5 (0.3)	-0.0155
Autoimmune disease	26 (2.1)	16 (0.9)	18 (1.4)	23 (1.4)	0.0063
Transplant (bone marrow/organ)	22 (1.8)	10 (0.6)	14 (1.1)	19 (1.1)	0.0049
Disability‡	28 (2.3)	23 (1.3)	21 (1.7)	26 (1.5)	0.0141

IQR, interquartile range; SD, standard deviation; SMD, standardized mean difference.

* Unknown refers to either one of, or both, race and ethnicity are unknown.

† Immunocompromised conditions included HIV/AIDS, solid malignancy, bone marrow transplant, organ transplant, rheumatologic/other inflammatory condition, primary immunodeficiency, chronic kidney disease/end stage renal disease, and other immune conditions.

‡ Includes neurologic, neurodevelopmental, intellectual, physical, vision or hearing impairment.
